# Research on the Methods and Algorithms Improving the Measurements Precision and Market Competitive Advantages of Fiber Optic Current Sensors

**DOI:** 10.3390/s20215995

**Published:** 2020-10-22

**Authors:** Valentina Temkina, Andrei Medvedev, Alexey Mayzel

**Affiliations:** Institute of Physics, Nanotechnology and Telecommunications, Peter the Great St.Petersburg Polytechnic University, 29 Polytechnicheskaya, 195251 St.Petersburg, Russia; temkina.vs@edu.spbstu.ru (V.T.); amayzel@gmail.com (A.M.)

**Keywords:** fiber optic current sensor, current measuring, Faraday effect, spun fiber, computer model

## Abstract

An electromagnetic instrument transformer is a common device used to measure large current values in high-voltage electrical networks; it has been in use for more than a century. However, the optical current transformer, a promising technology also known as a fiber optic current sensor (FOCS), offers increased safety and ease of operation, as well as the absence of errors caused by the magnetic circuit of legacy transformers. Although the FOCS scheme is well known and has been actively developed for over a quarter century, it has certain disadvantages that limit its use. This paper describes the authors’ efforts to solve these problems in order to make FOCS technology competitive and widely adopted. We upgraded the FOCS optical circuit, expanded the frequency band of the captured current signal, and reduced the solution’s cost. We designed new signal processing algorithms to compensate for errors caused by internal factors in the measurement circuit, as well as those caused by environmental influences. We developed an FOCS computer model based on the Jones matrix formalism to enhance the experimental debugging. It allowed us to define the requirements for elements of the optical circuit and its production accuracy.

## 1. Introduction

The widely adopted method to measure electric current at higher voltages is with an instrument transformer, a transducer converting primary current into proportional secondary current signals using two inductively coupled wire coils on a magnetic core. It is a legacy and an easy-to-implement way of transforming high current to low current that can be measured by instruments [1]. However, despite its simplicity and over a century of evolution, this device has significant and commonly known disadvantages. In instrument transformers, core magnetization is non-linear. As a result, major changes in the primary current signal during transients in the controlled network (starting currents, faults, etc.) cause saturation of the magnetic circuit, leading to a lack of information transmission about the primary current during the first periods of the emergency transient. This information is important for successful localization and elimination of the accident; however, the error of measuring current transformers reaches 90%. There is a high probability of the electrical breakdown of such transformers, which is associated with problems when ensuring high-voltage isolation. This leads to explosion and fire hazards. Finally, these transformers are of large dimensions and weights, and are also sensitive to electromagnetic interference [2,3,4,5,6,7].

The only solution to the issues mentioned above is a replacement of the physical phenomena that high current measurement devices rely on. The most likely replacement is polarized light radiation behavior in optical fiber placed in an electromagnetic field. The devices that implement this approach are known as fiber optic current sensors (FOCS).

This type of measurement devices has been a niche solution for over a quarter of a century. This is due to several weaknesses of FOCS technology [8,9,10,11,12,13,14,15,16,17,18,19]: accuracy drift over time and environmental conditions, output phase delay, difficulty in component production and assembly accuracy, and high overall capex. This paper describes the authors’ efforts to solve these problems and make FOCS technology competitive and preferable.

## 2. Fiber Optic Current Sensor Scheme

**Figure 1:**
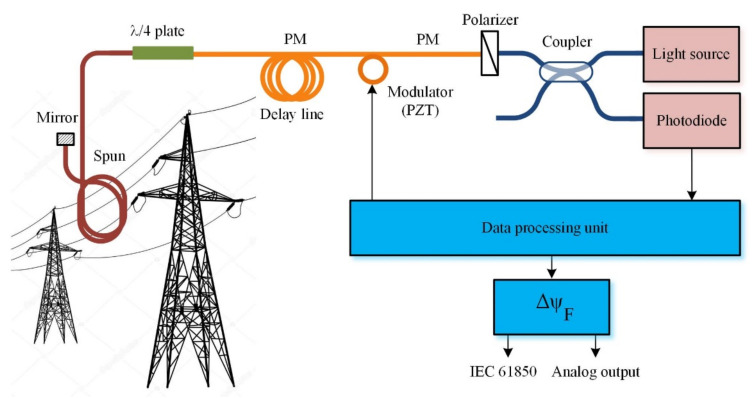
Optical scheme of fiber optic current sensor (FOCS).

The FOCS scheme is well-known and relies on the Faraday effect, which takes place in a special spun fiber wound around a current lead, affecting a polarized light phase (Figure 1). These FOCS are designed for voltage classes of 110–220 kV and higher; however, they can also be used at lower voltages.

The FOCS optical path consists of the following components:

- Light source
- Phase modulator made of a polarization-maintaining (PM) optical fiber coiled on a piezoelectric transducer (PZT)
- Fiber delay line
- Fiber quarter wave plate
- Sensing element made of spun optical fiber
- Mirror
- Polarizer
- Photodiode
- Data processing unit

In FOCS, a PZT resonant modulator is used for additional phase modulation of light by a harmonic signal with the following dependence:

(1) $$\Delta \psi_{M}\left(t\right)=\psi_{m}\cdot \cos\left(2\pi f_{M}t\right).$$

The use of such a modulator in the circuit limits the possible modulation frequency to the only available resonant frequency of piezoceramics (*f*_M_ = 40.1 kHz).

Phase detection is performed digitally. To do this, the first and second harmonics of the modulation frequency are extracted from the signal recorded by the photodetector and their ratio is analyzed [20]. This method is well-known in fiber optic gyroscopy and described in detail below.

### 2.1. Circuit Operation Description

Light radiation first propagates through the fiber optic coupler, then though the polarizer, and on to the phase modulator. The polarizer is rotated 45° against the proper polarization axes of the modulator’s PM fiber. The modulator’s output appears to be two orthogonally polarized light modes. The following delay line extends the light path length to meet the phase reverse requirements: the backward wave should be strictly 180° against the modulation phase. The scheme’s main enabling component is ¼ wave phase plate. It is again turned 45° to the proper axes of the upcoming path and performs transformation of the linearly polarized light to the circularly polarized modes. The circularly polarized light propagates through a sensitive element made of special spun fiber, which is wound around the conductor with the measured electric current. Therein, the light gains a phase shift, as shown below:

(2) $$\Delta \psi_{F}=2VNI,$$

where *V* is the fiber’s Verde coefficient, *N* is the number of fiber coil turns around the conductor with current *I* [10].

The circularly polarized light reflects from the mirror and propagates the full path backwards. The phase plate performs the reverse transformation of the circularly polarized modes into linearly polarized ones. The phase shift caused by Faraday effect in the sensitive fiber is kept in the signal. The modes with the linear polarization undergo interference in the polarizer and the interference signal gets captured by the photodiode after the splitter.

### 2.2. Signal Demodulation Algorithm

The optical power of the signal at the input of the photodiode is the sum of the harmonics of the modulation frequency:

(3) $$P\left(\Delta \psi_{F},\;t\right)=P_{0}\left[1+\cos\left(\Delta \psi_{F}+\psi_{m}\cos\left(2\pi f_{M}t\right)\right)\right]=\;=P_{0}\left[1+\cos\left(\Delta \psi_{F}\right)\cos\left(\psi_{m}\cos\left(2\pi f_{M}t\right)\right)-\sin\left(\Delta \psi_{F}\right)\sin\left(\psi_{m}\cos\left(2\pi f_{M}t\right)\right)\right].$$

Using expansion in terms of Bessel functions gives:

(4) $$P\left(\Delta \psi_{F},\;t\right)=P_{0}+P_{0}\cos\left(\Delta \psi_{F}\right)\;o\cdot \left[J_{0}\left(\psi_{m}\right)+2{\;J}_{2}\left(\psi_{m}\right)\cos\left(4\pi f_{M}t\right)+\;2{\;J}_{4}\left(\psi_{m}\right)\cos\left(8\pi f_{M}t\right)+\ldots \right]+\;+P_{0}\sin\left(\Delta \psi_{F}\right)\left[2{\;J}_{1}\left(\psi_{m}\right)\sin\left(2\pi f_{M}t\right)+2{\;J}_{3}\left(\psi_{m}\right)\sin\left(6\pi f_{M}t\right)+\ldots \right].$$

where in $J_{k}\left(\psi_{m}\right)$ are Bessel functions of the first kind of the k-th order.

Equation (4) shows the dependency of the harmonics’ amplitudes on optical power $P_{0}$ and the Bessel functions’ values. The even harmonics depend on the $\cos\left(\Delta \psi_{F}\right)$ function, and the odd ones on $\sin\left(\Delta \psi_{F}\right)$. In the idle state, the signal only consists of even harmonic components, and the odd components appear in the presence of a current-related magnetic field.

**Figure 2:**
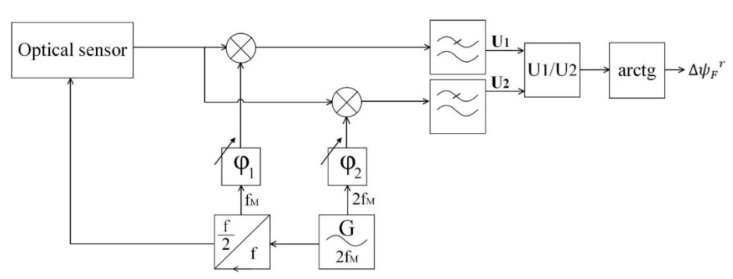
Block diagram of the signal demodulation method.

The signal processing method is universally adopted in fiber optic gyroscopes technology and relies on synchronous extraction of the first and second harmonics of the master frequency and the computation of their relationship (Figure 2).

We applied the synchronous detection of two tones of the captured signal (*f*_M_ and 2*f*_M_) to obtain a pair of quadrature signals:

(5) $$U_{1}\sim \sin\left(\Delta \psi_{F}\right)\cdot J_{1}\left(\psi_{m}\right)\cdot \cos\left(\Delta \varphi_{1}\right),$$

(6) $$U_{2}\sim \cos\left(\Delta \psi_{F}\right)\cdot J_{2}\left(\psi_{m}\right)\cdot \cos\left(\Delta \varphi_{2}\right),$$

where $\varphi_{1}$ and $\varphi_{2}$–phase shifts between reference synchronous signals and the extracted harmonic components. We used digital phase pre-sift to reach the equality $\cos\left(\Delta \varphi_{1}\right)=\cos\left(\Delta \varphi_{2}\right)=1$. Signal modulation amplitude was adjusted to satisfy the requirements of Bessel functions $J_{1}\left(\psi_{m}\right)$ and $J_{2}\left(\psi_{m}\right)$ to be equal and as big as possible.

The FOCS signal, thus, is described by the following equation:

(7) $$\Delta \psi_{F}{}^{r}=arctg{\left.\left[\frac{J_{2}\left({\psi_{m}}^{\prime }\right)}{J_{1}\left({\psi_{m}}^{\prime }\right)}\cdot \frac{U_{1}\left(t\right)}{U_{2}\left(t\right)}\right]\right|}_{\psi_{m}={\psi_{m}}^{\prime }}=arctg\left[tg\left(\Delta \psi_{F}\right)\right]=\Delta \psi_{F},$$

where $\Delta \psi_{F}$ and $\Delta \psi_{F}{}^{r}$—measured and calculated Faraday phase shifts, $\psi_{m}$ and ${\psi_{m}}^{\prime }$—measured and calculated modulation amplitude, and $U_{1}$ and $U_{2}$—the first and second tones of the master frequency.

### 2.3. Fiber Optic Current Sensor Errors

Despite two decades of market presence, the FOCS did not succeed in competing against legacy current transformers [8,9,10,11,12,13,14,15,16,17,18,19]. This is a result of imperfection in device design and signal processing algorithms. Engineers and researchers have been trying to improve the precision and robustness of the devices and lower their costs, investigating and bypassing the imperfections in data processing algorithms and the optical circuit.

The following disadvantages in the traditional FOCS scheme lead to measurement errors:

- Random deviation of the phase modulation value from its optimal point
- Temperature dependence of the quarter wave plate
- Phase error of the output signal
- Polarizing mismatches in the point of splicing of fibers and connections of fiber-optic elements
- Incorrect production of a quarter-wave plate

Each factor affects FOCS measurement error in its own way. By changing one factor, we investigated its effect and developed methods to eliminate the issue.

## 3. Assembling a Laboratory Prototype of the FOCS

We assembled an FOCS laboratory prototype that allowed us to solve several tasks in a bid to improve precision and accuracy, as well as lower the cost of goods in the circuit assembly.

The FOCS scheme from Section 2 has a delay line produced out of expensive PM fiber with length of approximately 1 km. It depends on the master frequency of modulation *f*_M_ as follows:

(8) $$l=\left(\frac{\frac{1}{2f_{M}}}{\tau }\right)/2,$$

where τ=5 us/km—time of light propagation in media.

Traditionally used PZT-based phase modulators (piezoelectric transducer) are resonant by nature and strictly determine the master frequency at 40 kHz. This frequency requires the circuit length to be 1250 m. To shorten the path required for operation, it is imperative to increase the master frequency of the modulator, which is unachievable using a static-frequency resonant component. Thus, we replaced the piezoelectric transducer with an electro-optical modulator (EOM), based on the Pockels effect. This type of component is suitable for up to gigahertz frequency ranges and permits consideration of other factors to determine proper frequency, like ADC rate or photodetector bandwidth. Increasing the frequency by 10 times reduces the required length of the optical path by the same 10 times (e.g., 400 kHz modulation requires 125 m of path length).

**Figure 3:**
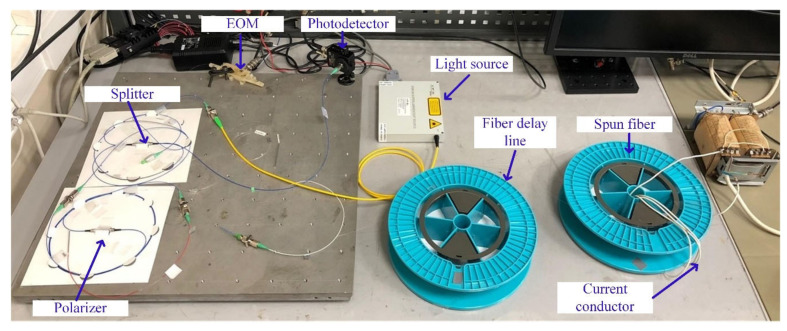
Laboratory model of FOCS.

**Figure 4:**
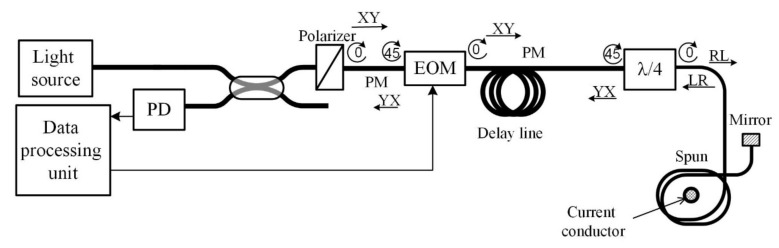
Optical scheme of the laboratory model of FOCS.

An FOCS laboratory prototype is shown in Figure 3; the optical scheme is given in Figure 4.

The main components of the FOCS prototype are:

- Erbium fiber superluminescent source ESS-30-M-01; nominal output power is 30 ± 3 mW, working wavelength is 1550 nm, width of the radiation spectrum at the level of -4 dB is not less than 20 nm
- 2 × 2 fiber splitter PFC-15-2-50-BB, which passes both fast and slow modes
- Fiber polarizer ILP-15-1-SA; skips the slow mode and blocks the fast one
- Electro-optical modulator; the half-wave voltage to modulate the polarization state is 4.9 V
- Delay line made of birefringent fiber HB1250 of the Bow-Tie type; the length of the polarization beats is 3.28 mm, the length of the fiber is 200 m
- Quarter wave plate made of the same fiber as the delay line
- The sensor element is a spun fiber coil SHB1500 (8.9/125); the length of the circular beats is 87.3 mm, the pitch of the spiral is 4.8 mm, spun fiber length is 90 m, radius of the coil is 8 cm, the coil contains 179 turns of fiber
- The right-angled end of the spun fiber is used as a reflector, which provides a reflection coefficient of about 4%
- Four turns of copper wire were passed through the spun fiber coil, along which the measured current was flowing
- PDA10CS-EC photodetector; a maximum bandwidth is 17 MHz at a gain of 0 dB.

There is the requirement for the light wave to arrive at the opposite phase against modulation. Thus, for the mock-up assembled with the total PM+Spun fiber length of 290 m according to Equation (8), the master frequency should equal 172.144 kHz. The FPGA implementation, for its part, makes it convenient to select a master modulation frequency from the discrete list formed by integer divisors and multipliers applied to the FPGA base clock. The base clock of the FPGA in the laboratory mock-up was set to 100 MHz and the data buffer was defined at 2000 points, resulting in the closest to the optimal available master frequency of 150 kHz.

The authors implemented the FOCS prototype software in the NI LabVIEW graphical programming environment. Registration and signal generation was performed using a PXI FPGA module for FlexRIO PXIe-7966 with an adapter transceiver module for FlexRIO NI-5783 manufactured by National Instruments, installed in the NI PXIe-1071 chassis.

**Figure 5:**
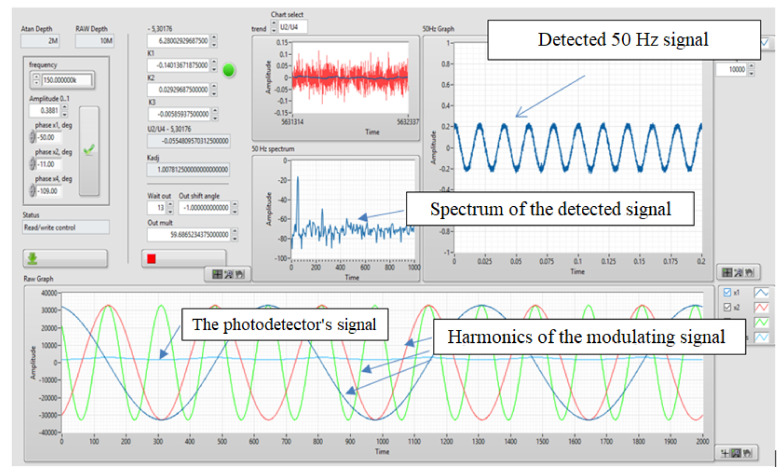
Front panel of the FOCS signal processing program in LabVIEW.

The front panel of the FOCS signal processing program is shown in Figure 5.

## 4. Measurement Compensation Methods and Results

### 4.1. Compensation of Measurement Dependence on Modulation Amplitude

Equation (7) shows that the results of the demodulation algorithm will strictly match the real measured value only under conditions of perfect junction alignment and optimal modulation value. However, all these conditions are just idealization and the junction mismatch in real circuit will introduce a constant error, and the value of modulation amplitude will vary over time with the changing environment conditions adding an unpredictable error. There are problems sourcing from these circumstances: the optimal modulation amplitude should be determined and strict requirements on the elements’ junction precision should be set.

We based on the following while determining the optimal modulation amplitude:

- The first *U*_1_ and second *U*_2_ tones of the master frequency should be equal and maximum possible to fit the ADC range better
- Optimal amplitude should represent the point of maximum sustainability, meaning the lowest possible error derivative over amplitude variations

The optimal value of modulation amplitude $\psi_{m0}$ = 2.630 radians was calculated. This value meets the requirements of $J_{1}\left(\psi_{m0}\right)=J_{2}\left(\psi_{m0}\right)$.

If the modulation amplitude differs from this value, the ratio of the Bessel functions in Equation (7) is not equal to 1, introducing an error in the processing result. To solve this issue, the authors developed a compensation method allowing to keep the Bessel functions ratio in Equation (7) equal to 1 in a wider range of amplitudes. They managed to determine the smaller amplitude deviations in real time based on the ratio of the second and fourth tones of master frequency in the captured signal:

(9) $$\frac{U_{2}}{U_{4}}=J_{2}\left(\psi_{m}\right)/J_{4}\left(\psi_{m}\right).$$

Equation (7) with compensation included looks as follows:

(10) $$\Delta \psi_{F}{}^{r}=arctg\left[\frac{U_{1}}{U_{2}}\cdot \left\{1+k_{1}\left(\frac{U_{2}}{U_{4}}-K_{0}\right)+k_{2}{\left(\frac{U_{2}}{U_{4}}-K_{0}\right)}^{2}+k_{3}{\left(\frac{U_{2}}{U_{4}}-K_{0}\right)}^{3}\right\}\right]=\;=arctg\left[\frac{J_{1}\left(\psi_{m}\right)}{J_{2}\left(\psi_{m}\right)}\cdot g\left(\psi_{m}\right)\Delta tg\left(\cdot \psi_{F}\right)\right],$$

where $U_{1}$, $U_{2}$, $U_{4}$—the harmonics of the master frequency, $k_{1}$, $k_{2}$ and $k_{3}$—compensating coefficients, $g\left(\psi_{m}\right)$—error correction function

(11) $$g\left(\psi_{m}\right)=1+k_{1}\left(\frac{J_{2}(\psi_{m})}{J_{4}(\psi_{m})}-K_{0}\right)+k_{2}{\left(\frac{J_{2}(\psi_{m})}{J_{4}(\psi_{m})}-K_{0}\right)}^{2}+k_{3}{\left(\frac{J_{2}(\psi_{m})}{J_{4}(\psi_{m})}-K_{0}\right)}^{3},$$

(12) $$K_{0}=\frac{J_{2}(\psi_{m0})}{J_{4}(\psi_{m0})}=5.302.$$

**Figure 6:**
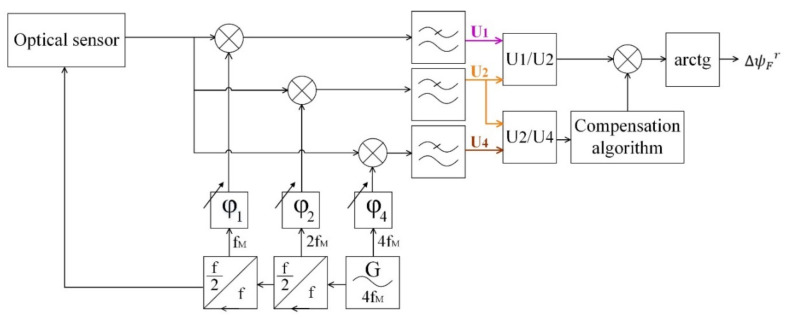
Block diagram of signal demodulation with compensation.

The modified block diagram of signal demodulation looks like in Figure 6.

We implemented the method described above to the mock-up in the FPGA and Real-Time code, extracting in additions to the first two harmonics the fourth one and adding a code applying the compensation to the demodulation process.

**Figure 7:**
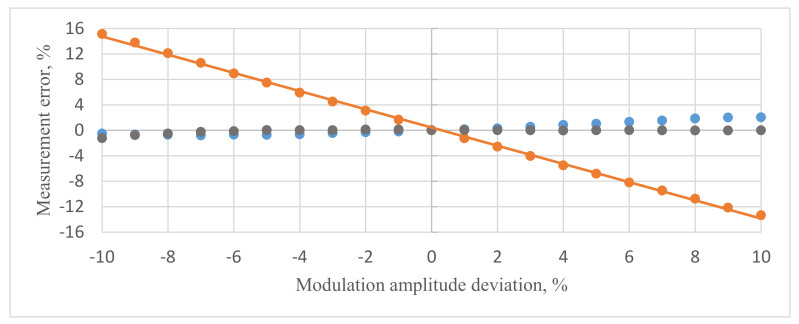
Measurement error vs modulation amplitude deviation of the optimal value in the range of ±10%. Blue—compensation with coefficients during numerical simulation, orange—without compensation, black—compensation with experimentally selected coefficients.

The laboratory mock-up passed a series of tests to verify compensation algorithm effectiveness. The first test (orange dots in Figure 7) was conducted without the compensation algorithm in signal processing. The authors changed the modulation amplitude ψ_m0_ in range ±10% of the optimal point and registered the FOCS error with an industrial comparator instrument used in power grid for measurement transformers regular verification. This device measured the deviation of actual current amplitude and phase with the nominal values of standard current transformer by the differential method. Its nominal current is I_nom_ = 5 A, and the actual current level during the experiments was half of this range (precisely 51.1%). The orange dependence in Figure 7 demonstrated that the FOCS measurement error increased by more than 15% when the modulation amplitude was ±10% of the optimal point.

The second test (blue dots in Figure 7) was conducted with the compensation algorithm, applying Equation (10) with compensation block (11). The coefficients $k_{1}$, $k_{2}$, and $k_{3}$ were identified using a computer simulation model and published in [21]. The values calculated were: $k_{1}=-0.162$, $k_{2}=0.038$, and $k_{3}=-0.005$. The modulation amplitude was changed in the same range. The blue dependence in Figure 7 demonstrated that the FOCS measurement error was as low as ±2.5% when the modulation amplitude was 10% of the optimal point.

However, the authors found that compensation could be improved. They specified the coefficients relying on experimental results rather than on the computer simulation. The refined values were $k_{1}=-0.140$, $k_{2}=0.029$, $k_{3}=-0.006$. The black dependence in Figure 7 showed that the use of these coefficients lowered the FOCS measurement error to ±1% in the same range of modulation amplitude deviation.

**Figure 8:**
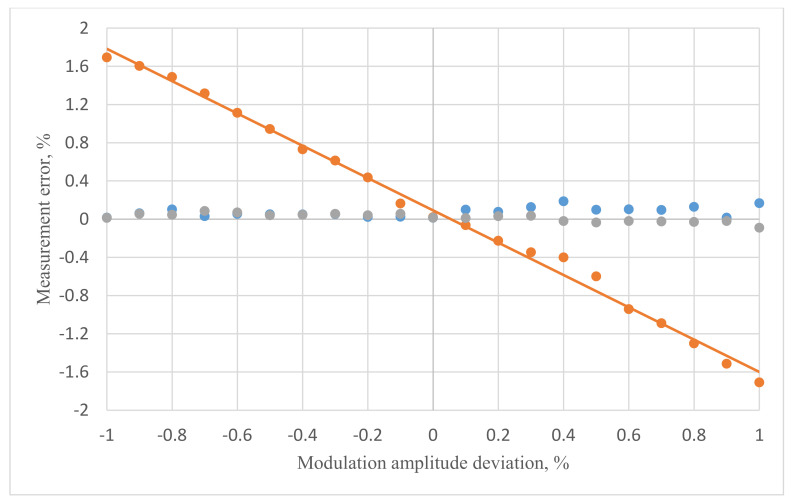
Measurement error vs modulation amplitude deviation of the optimal value in the range of ±1%. Blue—compensation with coefficients during numerical simulation, orange—without compensation, black—compensation with experimentally selected coefficients.

Further, we conducted a series of more detailed studies of FOCS error behavior in the range of uncontrolled deviation of modulation amplitude by ±1%. This can be caused by various external factors. The resulting dependencies were shown in Figure 8. The orange dots demonstrated the growth of FOCS error without compensation algorithm during signal processing. The blue dots demonstrated the error behavior when applying the compensation algorithm with coefficients obtained by numerical modeling. In addition, the black dots demonstrated the behavior of the FOCS error when applying the compensation algorithm with coefficients obtained experimentally.

Using the processing method without the compensation algorithm resulted in ±1.8% of error in ±1% range of the optimal modulation value. Using the refined coefficients in the compensation algorithm made the system accuracy stay in the margins of ±0.1%, which met the most severe industrial requirements.

### 4.2. Quarter Wave Plate Thermal Compensation

The other source of additional error is the temperature dependence of the delicate optical components of the circuit. The most susceptible component to temperature variations is the ¼-wave plate made of special fiber. Its optical length should be extremely precise to enable overall measurement precision; however, in terms of physical length, it should be just a couple of millimeters long, depending on fiber type. We introduced a method of component production that is repeatable and precise enough to satisfy industry needs [22].

**Figure 9:**
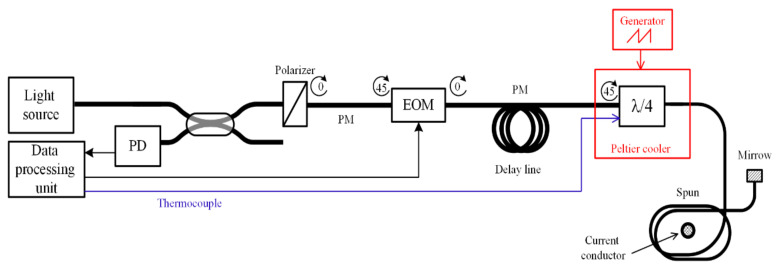
Experimental circuit for research on the effect of the ¼-wave plate temperature on FOCS error.

To isolate the influence of temperature of the ¼-wave plate temperature, the testbed shown in Figure 9 was assembled. The plate was covered in a thermal chamber with a Peltier element, allowing it to heat and cool the inside volume. During the approximately 30 min, the chamber was first heated to about 53 °C and then cooled to 15 °C. The same industrial comparator instrument was used to capture the error value.

**Figure 10:**
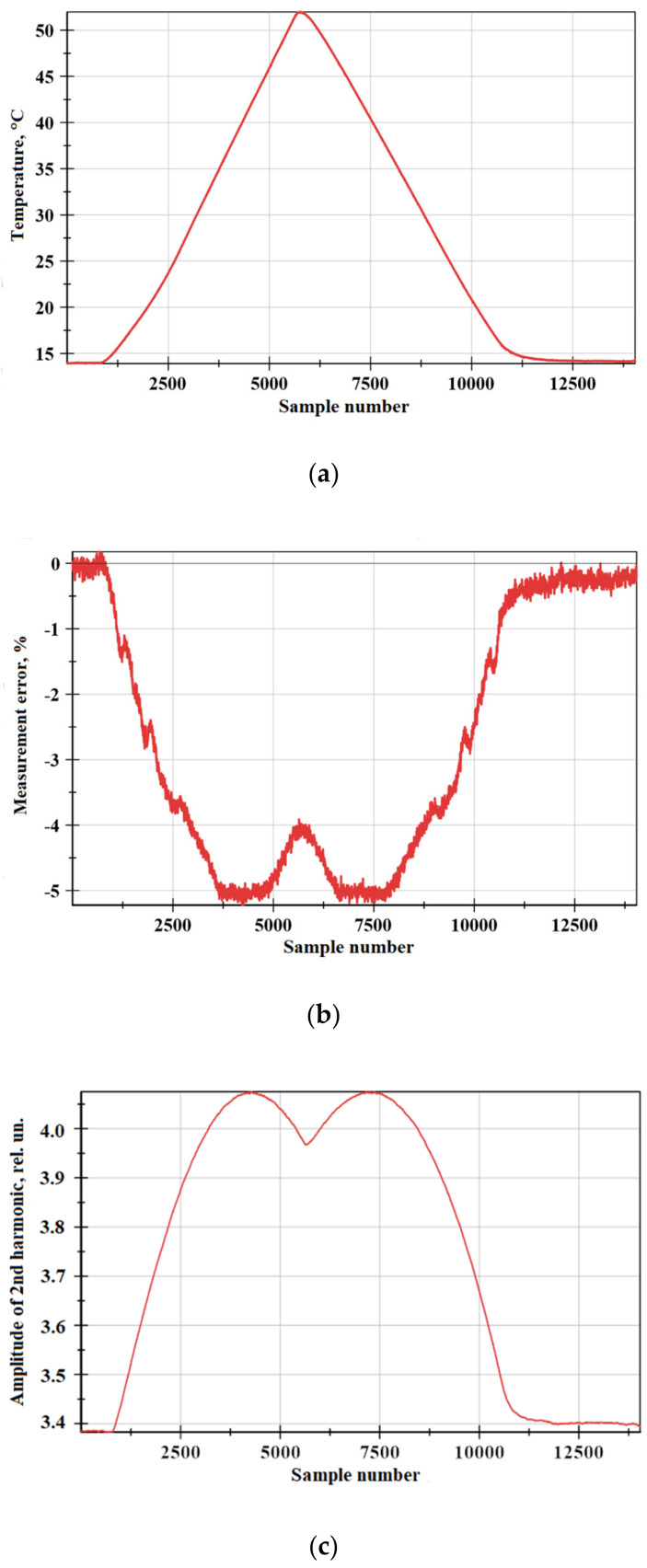
The experimental results: (**a**) Quarter-wave plate’s temperature vs. sample number; (**b**) relative amplitude error of FOCS vs. sample number; (**c**) amplitude of second harmonic vs. sample number. The primary current was 50% of the nominal value.

The research results are shown in Figure 10. It can be seen that as the temperature of the ¼-wave plate changes, the FOCS error increases, and the nature of this dependence is neither linear nor monotonic. In Figure 10b, certain decreases in measurement error can be found at around the temperature peak. We assume that the possible reason for such tendencies may be the periodic dependence of the fiber plate length (in terms of λ, i.e., 1/4λ, 1/2λ, 3/4λ, λ) on the temperature. Changing the temperature of the fiber phase plate leads to its optical length changing, which means that it may no longer be a quarter-wave. As the temperature increases, it turns into a half-wave plate at a certain point. In this case, we should expect the largest FOCS measurement error. With a further increase in temperature, the error will decrease as long as the fiber plate length tends to be 3/4λ. After this point, the error is expected to grow, but with the opposite sign, until the plate length reaches λ. Such big changes are not achieved in optical length; sample numbers from 0 to 5500 correspond to a part of this periodical dependence, while heating of the plate and sample numbers from 5500 to 11000 are its cooling. It should be noted that these assumptions require additional research.

Moreover, this research demonstrates that the graph of the measurement error vs temperature of the ¼-wave plate exactly coincides with similar dependence of the amplitude of the second tone of the master frequency (compare Figure 10b and mirrored Figure 10c).

The apparent idea is to perform a temperature compensation on the error deviation. It appears to be an ideal solution for laboratory research due to the availability of accurate temperature measurement equipment, low external interference, and lack of industrial insolation requirements. Nevertheless, this approach is complicated and costly in industrial high-voltage applicable FOCS design. It would require an additional optical fiber with an FBG sensor and an expensive interrogator device, with the goal of no copper wire to appear in the optically isolated high-voltage area.

Instead of implementing direct temperature measurements in the high-voltage zone, we used the conformity of FOCS measurement error vs temperature and higher harmonics amplitude vs temperature graphs. Monitoring of higher tones requires no additional measurement channels, except for the main signal and inline digital signal processing algorithm. This processing was implemented in real-time and FPGA subsystems, delivering a compensation coefficient to the resulting output signal without additional delay.

**Figure 11:**
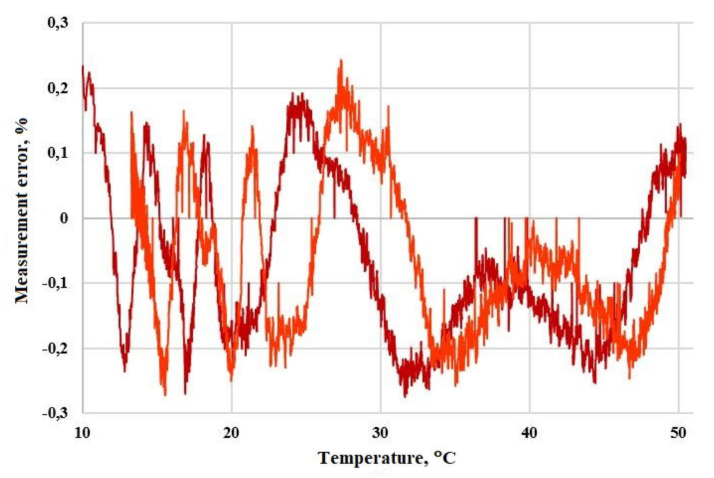
Relative amplitude error of FOCS after temperature compensation vs. temperature of the quarter-wave plate. Different colors correspond to heating and cooling. The primary current was 50% of the nominal value.

The study of the resulting system in the same conditions as before showed a dramatic decrease in FOCS measurement error vs temperature. This dependence is presented in Figure 11. Compared to the uncompensated output in Figure 10, it achieves about 10 times less error (up to 5% in Figure 10b and ±0.25% in Figure 11).

The obtained accuracy (as in Figure 11) is enough for current transformers of accuracy class 0.5 (i.e., ±0.5%). However, we would like to meet accuracy class 0.2. Hence, the target value on the measurement error to be achieved is ±0.2%. Although we eliminated the errors described above, repeated artefacts can still be seen. Therefore, it is necessary to identify its source. In addition, there is a saturation of error graph suspicion, which also needs to be investigated. It can be caused by many factors, such as external noise, imperfections of optical scheme, polarizing mismatches, and demodulation errors. Thus, it is necessary to exclude each of these effects to improve sensor accuracy and stability. Our research demonstrated that the meter measurement accuracy was influenced by a set of external factors, and it is difficult to study the system response to each factor individually in the real prototype. Moreover, it is expensive to debug it experimentally. In turn, a computer model of the FOCS will allow us to study the influence of many parasitic factors separately, as well as to check-out the signal processing algorithms, which is also necessary to provide real time high-precision measurements.

## 5. Modeling Method of the Fiber Optic Current Sensor

**Figure 12:**
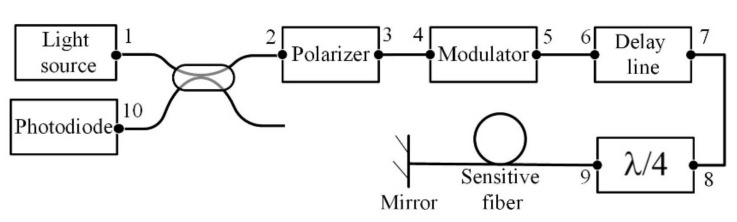
FOCS optical scheme in general form. The numbers 1–10 indicate the junctions between optical elements or fiber splicing.

Modeling of the FOCS was based on the formalism of Jones matrices. According to this method, each element of the optical circuit can be represented by a 2 × 2 matrix describing the transformation of the polarization state of light when passing through this element [23]. The FOCS optical scheme in general form is shown in Figure 12.

The modulator and the quarter-wave plate was represented by matrices of phase plates on the basis of linear polarizations, according to the following equation:

(13) $$\left[K_{n}\right]=\left[K\left(\varphi_{n}\right)\right]=\left[\begin{array}{cc} e^{-j\varphi_{n}/2} & 0\\ 0 & e^{j\varphi_{n}/2} \end{array}\right],$$

where *φ_n_* is the phase difference between the polarization modes formed during propagation through the element. Phase plate matrices for different elements had the same structure, but differed in phases. In the modulator, the phase shift consisted of two terms, according to Equation (13). The first random slowly changing value *φ*^0^_mod_ represented the quasi-static phase difference between the two polarization modes, and the second was variable and determined by the modulating signal *U*_mod_(*t*):

(14) $$\varphi_{\mathrm{mod}}=\varphi_{\mathrm{mod}}^{0}+k_{U}U_{\mathrm{mod}}\left(t\right).$$

The quarter-wave plate introduced a phase difference between the two polarization components, equal to:

(15) $$\varphi_{\lambda /4}=\frac{\pi }{2}+\pi m,$$

where *m* is an integer.

Rotation matrices were introduced to account for the rotation of coordinate axes to any element of the optical scheme relative to another element, on the basis of Equation (15):

(16) $$\left[R_{n}\right]=\left[R\left(\alpha_{n}\right)\right]=\left[\begin{array}{cc} \cos\alpha_{n} & -\sin\alpha_{n}\\ \sin\alpha_{n} & \cos\alpha_{n} \end{array}\right],$$

where *α_n_* is the angles at points where the fibers connect to each other or to elements. It should be noted that on the basis of linear polarizations, the sensitive fiber was also described by a rotation matrix. Since the plane of linear polarization of light turned at an angle $\varphi_{F}/2$ proportional to the current due to the Faraday Effect, which was identical to the change in $\varphi_{F}$ in the phase difference between light modes of circular polarization.

A polarizer transmitted only one polarization, which coincided with its own axis; in the ideal case, the matrix of the polarizer had the following form:

(17) $$\left[P\right]=\left[\begin{array}{cc} 1 & 0\\ 0 & 0 \end{array}\right].$$

When the light falls directly on the mirror and is then reflected off it, the direction of light propagation changes to the opposite. Accordingly, the mirror matrix must be such that a right-handed coordinate system is preserved. To do so, it was necessary to maintain the direction of one of the transverse axes and change the direction of the other to the opposite. Thus, the mirror matrix was a half-wave plate matrix [24]. Let the x-axis retain the direction, and y-axis be changed by 180°; then, the mirror matrix can be written as:

(18) $$F_{mir}=\left[\begin{array}{cc} 1 & 0\\ 0 & -1 \end{array}\right].$$

In addition, when the light is propagated in the opposite direction through the elements, their matrices must be modified. If the matrix of an reciprocal element in a forward direction was described by this equation:

(19) $$B=\left[\begin{array}{cc} b_{11} & b_{12}\\ b_{21} & b_{22} \end{array}\right],$$

then in the reverse direction of light propagation through this element, its matrix has the following form [24]:

(20) $$\bar{B}=\left[\begin{array}{cc} b_{11} & -b_{21}\\ -b_{12} & b_{22} \end{array}\right].$$

The fiber delay line in the optical circuit of the FOCS must provide such a delay between two orthogonally polarized modes that when light is propagated from the modulator to the mirror and back, the phase of the modulating voltage changes sign to the opposite. Therefore, in the case of an ideal delay line, its influence was directly taken into account in the modulator matrices.

Thus, sequentially multiplying the matrices of each optical element in the order opposite to the direction of light propagation in the scheme, we obtained a common matrix of the system:

(21) $$T=\bar{R_{10}}\cdot \bar{R_{2}}\cdot \bar{P}\cdot \bar{R_{3}}\cdot \bar{R_{4}}\cdot \bar{M}\cdot \bar{R_{5}}\cdot \bar{R_{6}}\cdot \bar{F_{dl}}\cdot \bar{R_{7}}\cdot \bar{R_{8}}\cdot \bar{F_{\lambda /4}}\cdot \bar{R_{9}}\cdot \bar{S}\cdot F_{mir}\cdot S\cdot R_{9}\cdot F_{\lambda /4}\cdot \;\cdot R_{8}\cdot R_{7}\cdot F_{dl}\cdot R_{6}\cdot R_{5}\cdot M\cdot R_{4}\cdot R_{3}\cdot P\cdot R_{2}\cdot R_{1},$$

wherein [*R*_1–10_] is the rotation matrix by angle α_1–10_, [*P*] is the polarizer matrix, [*M*] is the modulator matrix, [*F_dl_*] is the delay line matrix, [*F*_λ/4_] is the quarter-wave plate matrix, [*S*] is the sensor matrix, [*F_mir_*] is the mirror matrix. The numbers 1-10 indicated the junction of optical elements or fiber splicing in accordance with Figure 12.

When all the optical elements were ideal and α_1_ = α_2_ = α_4_ = α_5_ = α_6_ = α_7_ = α_10_ = 0°, α_3_ = α_8_ = 45°, α_9_ = −45°, the common matrix of the system [*T*] was determined by the following equation:

(22) $$\left[T\right]=\left[\begin{array}{cc} 1 & 0\\ 0 & 0 \end{array}\right]\cdot \left[\begin{array}{cc} \cos45^{{}^{\circ}} & -\sin45^{{}^{\circ}}\\ \sin45^{{}^{\circ}} & \cos45^{{}^{\circ}} \end{array}\right]\cdot \left[\begin{array}{cc} e^{\frac{1}{2}j\varphi_{mod}} & 0\\ 0 & e^{-\frac{1}{2}j\varphi_{\mathrm{mod}}} \end{array}\right]\cdot \left[\begin{array}{cc} \cos45^{{}^{\circ}} & -\sin45^{{}^{\circ}}\\ \sin45^{{}^{\circ}} & \cos45^{{}^{\circ}} \end{array}\right]\cdot \;\cdot \left[\begin{array}{cc} e^{-\frac{j\pi }{4}} & 0\\ 0 & e^{\frac{j\pi }{4}} \end{array}\right]\cdot \left[\begin{array}{cc} \cos45^{{}^{\circ}} & \sin45^{{}^{\circ}}\\ -\sin45^{{}^{\circ}} & \cos45^{{}^{\circ}} \end{array}\right]\cdot \left[\begin{array}{cc} \cos\frac{\varphi_{F}}{2} & \sin\frac{\varphi_{F}}{2}\\ -\sin\frac{\varphi_{F}}{2} & \cos\frac{\varphi_{F}}{2} \end{array}\right]\cdot \left[\begin{array}{cc} 1 & 0\\ 0 & -1 \end{array}\right]\cdot \;\cdot \left[\begin{array}{cc} \cos\frac{\varphi_{F}}{2} & -\sin\frac{\varphi_{F}}{2}\\ \sin\frac{\varphi_{F}}{2} & \cos\frac{\varphi_{F}}{2} \end{array}\right]\cdot \left[\begin{array}{cc} \cos45^{{}^{\circ}} & \sin45^{{}^{\circ}}\\ -\sin45^{{}^{\circ}} & \cos45^{{}^{\circ}} \end{array}\right]\cdot \left[\begin{array}{cc} e^{-\frac{j\pi }{4}} & 0\\ 0 & e^{\frac{j\pi }{4}} \end{array}\right]\cdot \;\cdot \left[\begin{array}{cc} \cos45^{{}^{\circ}} & -\sin45^{{}^{\circ}}\\ \sin45^{{}^{\circ}} & \cos45^{{}^{\circ}} \end{array}\right]\cdot \left[\begin{array}{cc} e^{-\frac{1}{2}j\varphi_{mod}} & 0\\ 0 & e^{\frac{1}{2}j\varphi_{\mathrm{mod}}} \end{array}\right]\cdot \left[\begin{array}{cc} \cos45^{{}^{\circ}} & -\sin45^{{}^{\circ}}\\ \sin45^{{}^{\circ}} & \cos45^{{}^{\circ}} \end{array}\right]\cdot \left[\begin{array}{cc} 1 & 0\\ 0 & 0 \end{array}\right].$$

The common matrix of the system connected the output Jones vector, which described the state of light polarization at the output of the optical circuit, with the input Jones vector, which described the state of light polarization at the input of the optical circuit, according to this equation:

(23) $$\left[D_{\mathrm{out}}\right]=\left[T\right]\cdot \left[D_{\mathrm{in}}\right].$$

Then the intensity of the light beam was calculated as:

(24) $$I={\left[D_{out}^{*}\right]}^{T}\cdot \left[D_{out}\right].$$

Finally, the received intensity was transmitted to the signal processing unit.

## 6. Computer Simulation of the Fiber Optic Current Sensor and Its Results

### 6.1. Building of the Fiber Optic Current Sensor Model in LabVIEW

As the FOCS prototype control and processing algorithms were implemented in a LabVIEW programming environment, the decision was made to develop a simulation model using the same tools. LabVIEW is a graphical programming language that uses graphical images (icons) as functions, providing perfect clarity and traceability of code logic. This made LabVIEW a widely adopted tool for solving various scientific and engineering tasks, including the implementation of models of fiber-optic systems [25].

The simulation provided advantages such as mock-up code reuse, along with the ability to add, exclude, or replace various elements of the optical scheme without rewriting the program code. In addition, it is not always possible to analytically solve the influence of parasitic factors. Prototype measurement error and analysis are significantly complicated by the presence of all disturbing factors simultaneously. In this regard, the proposed FOCS simulation allows to significantly simplify the analysis. It does not require to derivate the analytical formula; only the parameters for the model are required to be set—for example, the frequency and amplitude of the modulation—as well as parameters for external influences.

**Figure 13:**
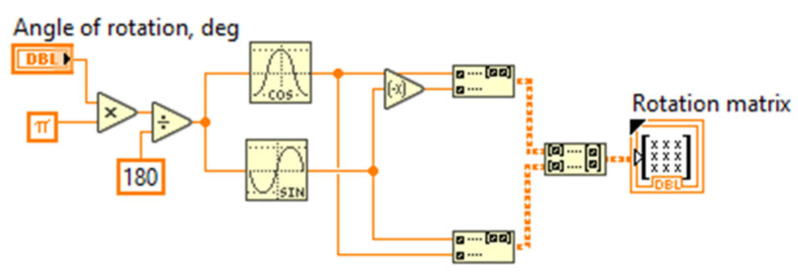
Program code in LabVIEW to form a rotation matrix.

Each matrix in Equation (21) was modeled as a separate virtual instrument (VI), stored in LabVIEW and being used in the common program for the FOCS model. Figure 13 shows the program code that forms the rotation matrix.

**Figure 14:**
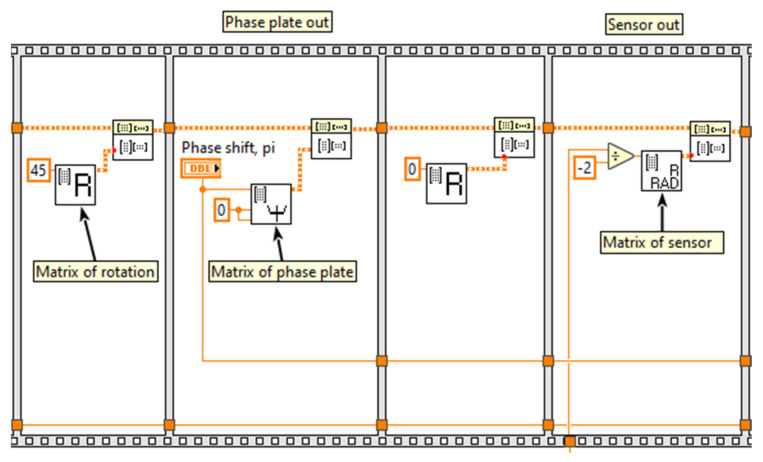
FOCS modeling in LabVIEW (the block diagram).

Figure 14 shows a part of the common program for the FOCS model. It demonstrates the matrix of circuit elements as VI and their connection in the common program. The advantages of this approach are visibility and the ability to add, exclude, or replace various optical elements without rewriting the program code, because the matrices of each element are located in separate frames.

**Figure 15:**
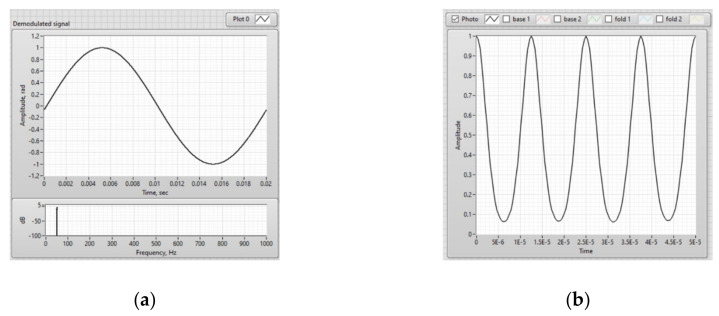
Results obtained during the FOCS modeling: (**a**) Demodulated signal and its spectrum; (**b**) Interference signal.

A harmonic signal with frequency of 50 Hz was set as a signal simulating an electric current and acting on a sensitive fiber. Thus, the real-time FOCS model generated a raw interference signal that arises at the output of the sensor optical circuit when magnetic field is exposed to the sensor fiber, as well as a demodulated signal that characterized this effect (Figure 15). This set output signal corresponds to the real laboratory prototype of the FOCS.

Based on Figure 15, it can be seen that the demodulated signal is identical to the measured harmonic current signal with a frequency of 50 Hz. The developed model, implemented in LabVIEW, describes the actual physical processes occurring in the FOCS. The result of the simulation is fully confirmed by measurements of the FOCS laboratory prototype. In addition, this model can be used for debugging FOCS signal processing algorithms, since it is optimized for both floating-point and fixed-point values, which is necessary for implementing the code on the FPGA.

The FOCS measurement error and its analysis are substantially complicated by the presence of all external parasitic factors at the same time. In this regard, the developed FOCS model simplifies the study. For example, we can add noise to the system, polarizing mismatches at the junctions of fibers, take into account the imperfection of the quarter-wave plate, and individually research the influence of each factor on measurement accuracy.

### 6.2. Influence of Polarization Mismatches on the Accuracy of Fiber Optic Current Sensor Measurements

The case of polarization mismatches occurrence in the circuit element located after the modulator and before the sensitive element, namely at point 8 of the FOCS optical circuit, was investigated. For this purpose, we assumed α_8_ ≠ 45° (Figure 12).

**Figure 16:**
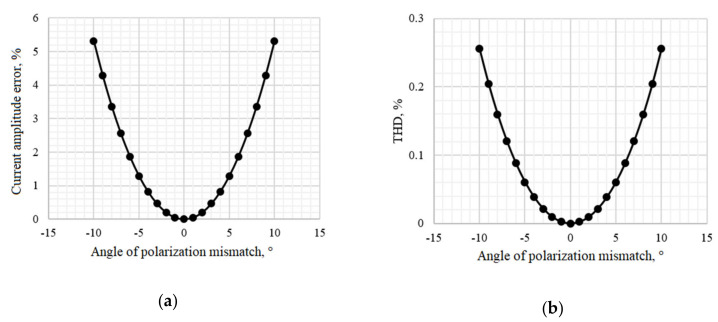
Dependences: (**a**) Current amplitude error vs. angle of polarization mismatch at point 8; (**b**) The total harmonic distortion vs. angle of polarization mismatch at point 8.

**Figure 17:**
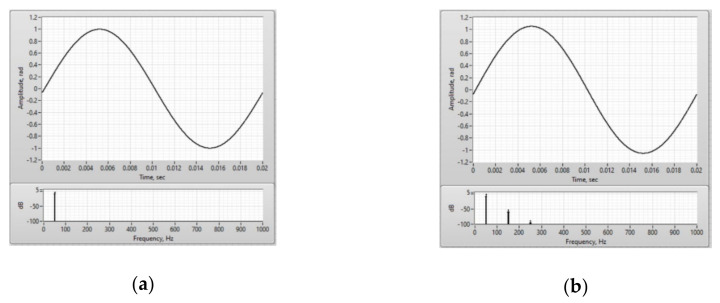
Demodulated current signal and its spectrum. The angle of polarization mismatch at point 8 is: (**a**) 0°; (**b**) 10°.

Using the developed model, the dependence of the measured current amplitude error and the total harmonic distortion on the angle of mismatch at point 8 was obtained (Figure 16). The demodulated signal and its spectrum in the ideal case and with polarization mismatch by 10 degrees is demonstrated in Figure 17.

This research showed that the permissible tolerance of polarization mismatches at one of the optical circuit points located after the modulator and before the sensitive fiber is ±2° to achieve FOCS accuracy class 0.2. In addition, the average level of the interference signal at the output of the optical circuit decreased by 9%, with a mismatch angle of 10°.

### 6.3. Influence of Quarter-Wave Plate Imperfections on the Accuracy of Fiber Optic Current Sensor Measurements

**Figure 18:**
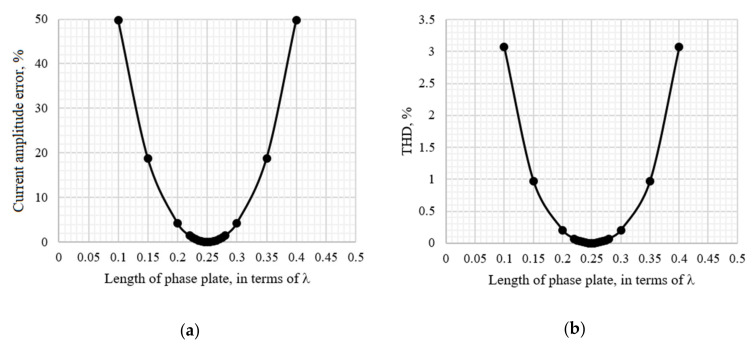
Dependences: (**a**) Current amplitude error vs. length of phase plate; (**b**) Total harmonic distortion (on the right) vs. length of phase plate.

**Figure 19:**
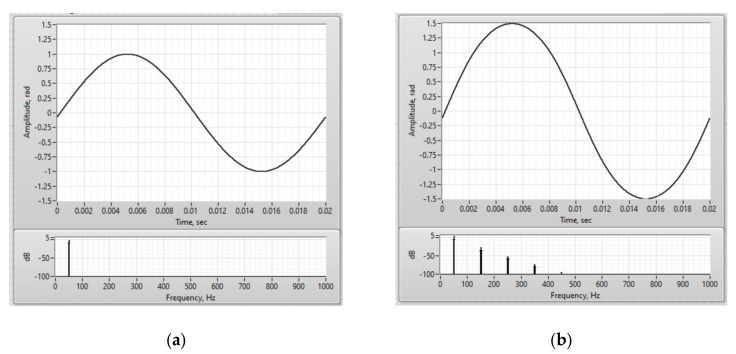
Demodulated current signal and its spectrum: (**a**) The case of ideal ¼-wave plate; (**b**) Phase plate with length of 0.1λ.

A ¼-wave plate was a small piece of birefringent fiber only a few millimeters in size, but the accuracy of the entire huge system depended on it. The research found that incorrect manufacturing of a ¼-wave plate, expressed in a mismatch in its length, led to a significant increase in current amplitude error and the appearance of nonlinear distortions (Figure 18 and Figure 19).

**Figure 20:**
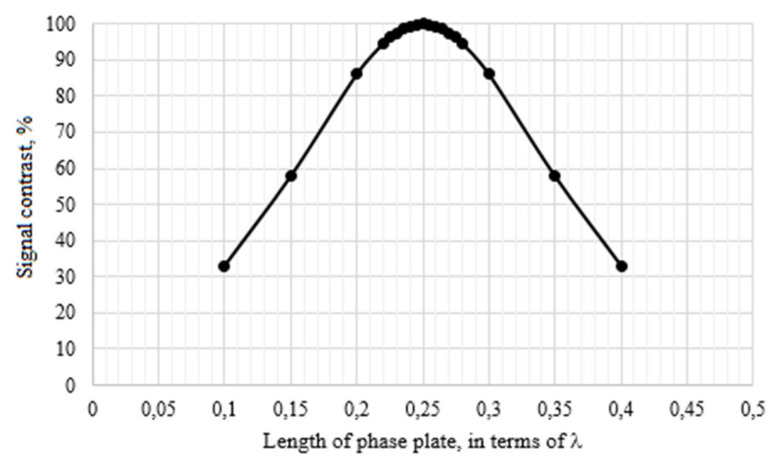
Contrast of the interference signal vs. length of the phase plate.

Differing from the effect of polarization mismatches at the points of element junctions or fiber splicing, the ¼-wave plate also affected the contrast of the interference signal at the output of the optical circuit (Figure 20). Signal contrast was calculated using the following equation:

(25) $$V=\frac{I_{max}-I_{min}}{I_{max}+I_{min}}\cdot 100\%,$$

where *I* is the signal intensity at the optical circuit output.

This shows that imperfect ¼-wave plates reduce the signal contrast and thereby limit the dynamic range of the current sensor. This is due to the fact that modes with elliptical polarization are formed at the output of the phase plate. These modes are not converted to orthogonal modes when reflected from a mirror. As a result, when light passes back through the phase plate, the output is again elliptically polarized modes, rather than linear ones [13].

Thus, the permissible tolerance of the error of the ¼-wave plate length is ±0.01λ to achieve FOCS accuracy class 0.2. In these error margins, the contrast of the interference signal is reduced by only 0.62%.

A similar study was conducted with the FOCS mock-up. The results of the experiments coincided with the simulation results. It confirmed the efficiency of the developed model and the relevance of its application in finding ways to eliminate the overall FOCS errors.

### 6.4. Research on the FPGA Algorithm of Signal Processing for Fiber Optic Current Sensors

The interference signal modeled on the output of the FOCS optical circuit corresponds to the signal received by the photodetector in the mock-up circuit shown in Figure 4. This signal is transferred to the signal-processing unit to extract the measured current and compensate the errors. In the laboratory prototype of the FOCS, we encountered the problem of unexpected artifacts that sometimes led to the failure of the scheme. It was a sudden process, the nature of which was difficult to determine intuitively, since it occurred under different circumstances. In addition, the study of this problem was difficult due to the hardware implementation of control and processing algorithms based on FPGA. It required careful processing of fixed and integer data types, avoiding data saturation and loss, and took about two hours of compilation time for each code change. However, no platform other than FPGA could provide nanosecond synchronization of modulating control and measurements with inline demodulation and error handling. Therefore, we should use in simulation the modeled interference signal at the output of the optical circuit instead of the real one for debugging of the simulation processing algorithms and detecting its limitations. This should allow us to reuse the FPGA code with simulated signals without any significant changes, then after applying new patches on it to move it back to FPGA seamlessly.

The simulation ran on 8th generation Core i7 mobile processor, with a time step of 1/2.56E6 s (1/10 of modulation frequency selected), at a speed of approximately 3.5 s per 0.02 s of the simulated process, which is 1 period of 50 Hz industrial current. It is not real time, but extremely more efficient than 2 h of FPGA code compilation. The best advantage of LabVIEW for modeling is that we moved the algorithms from FPGA code to execute on the host processor keeping the code “as is” with integer and fixed-point variables resolution. Unlike the compiled FPGA instance, the same code running on CPU is traceable, we can monitor, plot, or save to file every data value at every wire.

The demodulation algorithm worked with the simulation model exactly as we observed it to operate on physical mock-up. That was the approval of simulation model fidelity over its physical twin. Skipping the iterative process of algorithms polishing, we identified the limitations of phase signal demodulation.

**Figure 21:**
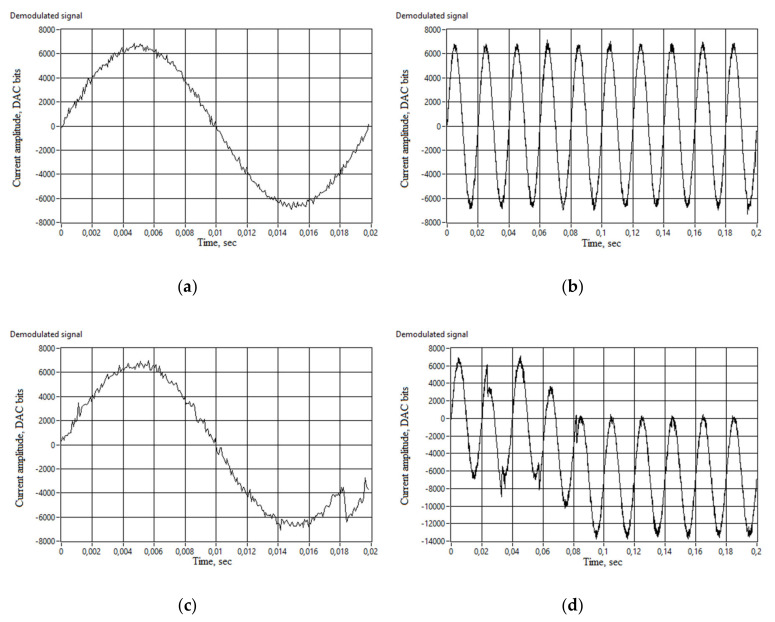
Demodulated current signal vs time: (**a**) Current signal of 2π radians, 20% noise, 1 period; (**b**) Current signal of 2π radians, 20% noise, 10 periods; (**c**) Current signal of 2π radians, 21% noise, 1 period; (**d**) Current signal of 2π radians, 21% noise, 10 periods.

Figure 21 demonstrates a difference between two settings: 2π current amplitude with 20% of noise and with 21% of noise in the measured current signal. It can be seen that in the first case, the signal is noisy but is a sine waveform of 50 Hz. Moreover, the noise is much less than 20% of the signal, due to averaging in the processing algorithm. Nevertheless, one more percent of noise does not make the demodulated signal worse. The algorithm completely fails. In this case, there are significant phase wraps that are unrecoverable with unwrap algorithm included in the processing code. Introducing more noise into the original current signal destroys the output, making no sine waveform discoverable.

The behavior described above is universal for this algorithm, but the threshold of 20% noise applies only to the current amplitude of 2π. There is probably a dependency over current vs. noise to demodulate efficiency of the algorithm.

**Figure 22:**
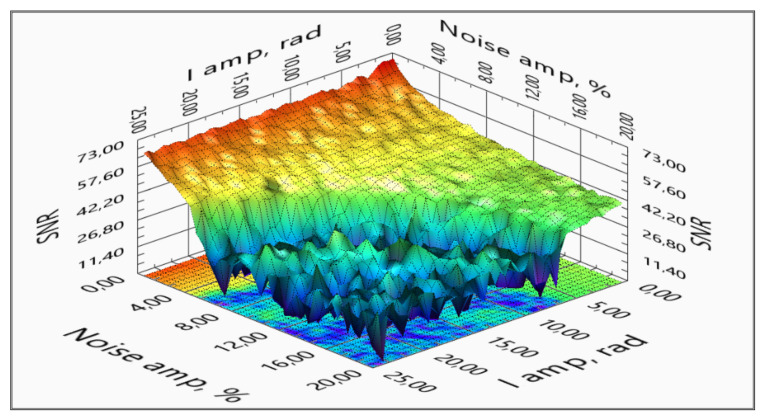
Measured signal SNR vs. signal amplitude vs. source signal noise.

**Figure 23:**
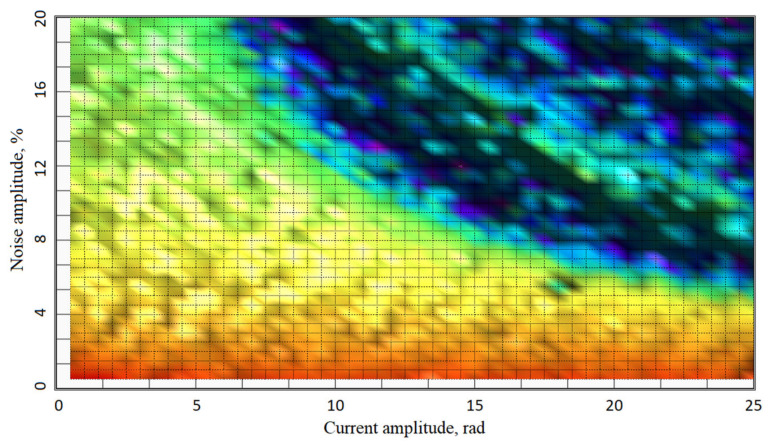
Current signal amplitude vs. noise % accuracy distribution.

Thus, we automated the discovery process, sending to the model a matrix of current amplitudes and noise percentage. The resulting SNR surface distribution and its projection are represented in Figure 22 and Figure 23. This shows that there is a threshold, a limit of algorithm robustness, where a minor disturbance makes the method inapplicable.

The projection in Figure 23 discloses the threshold trends. The higher the measured signal, the less robust is the algorithm against noise. Further research showed that at levels below π/2, the demodulation processing recovers the signal with 100% of noise, and at above 10π amplitude, the unwrapping of demodulated signal returns errors on 0.5% noise. This overall threshold line appears as a logarithmic graph with approximately 32 rad of X-axis intersection and, in theory, asymptotically approaching Y-axis, which is limited in real setup with LSB value of any ADC used. Fortunately, in real-world operation, the higher the current signal is, the lower noise percentage it contains. Moreover, the tolerance of the algorithm growth as the signal is lowered.

## 7. Conclusions

### 7.1. Research Result

FOCS technology has been on the market for several decades, but still faces a number of significant disadvantages that limits its scale of implementation. First, specialists face the challenge of improving the accuracy and reducing the cost of ready-made solutions.

We built an FOCS prototype, in which we replaced the piezoceramic modulator with an electro-optical one, thereby expanding the band of recorded frequencies and reducing FOCS cost, increasing the frequency of phase modulation to 150 kHz, and reducing the length of the expensive delay line to 200 m (6 times). By compensating for the effect of uncontrolled deviation of the modulation amplitude on the measurement results, we improved the accuracy and stability of the FOCS. Within the range of ±1% amplitude deviation, the measurement error did not exceed ±0.2%. In addition, we compensated for the effects of the quarter-wave plate temperature and other elements of the optical circuit on the measurement results without directly measuring the temperature and without including additional elements in the optical circuit. This made it possible to preserve the fully dielectric structure of the sensor element, as well as to increase the FOCS stability, while avoiding cost increases. Within the range of temperature changes from 15 °C to 53 °C, the FOCS measurement error did not exceed ±0.2%. The use of modern high-speed elements based on FPGA in the digital detection unit of the FOCS signal has made it possible to implement algorithms for processing of large data flows in real time and generate output signals with minimal delay.

Besides, the FOCS model was developed based on the Jones matrix formalism, instead of a physical object. Implementation of the model in the LabVIEW programming environment allowed us to study the sensor operation without analytical derivation of formulas. The developed model was a tool with which we were able to reduce the cost of finding and eliminating weaknesses that have a major impact on the parameters of the end device. Experiments showed that the model is a digital twin of the FOCS real prototype. The influence of polarizing mismatches in places of fiber splicing or junctions between optical elements, as well as the incorrectness of manufacturing a quarter-wave plate on the accuracy of current sensor measurements, was studied. When polarizing mismatches are introduced, the amplitude error of the current sensor and the non-linearity of the output signal increases. However, the imperfection of the quarter-wave plate also affects the contrast of the interference signal at the optical circuit output, and therefore the dynamic range of the device. In order for the FOCS to meet accuracy class of 0.2, it is necessary to ensure that the error of junctions between elements is not more than ±2° and the error of manufacturing a quarter-wave plate is not more than ±0.01λ. The results of the experiments with a real prototype coincided with the simulation results.

This research on the FPGA algorithm of signal processing for FOCS provides a great path for future research. The caveats and limitations are now visible, so they can be avoided. Similarly, perfect operation conditions with the widest dynamic range and best stability are now easily determinable to achieve the best accuracy and robustness of FOCS for industry implementation.

### 7.2. Research Prospects

Despite all the advantages and possibilities of the FOCS computer model, built on the basis of the Jones integral matrix formalism (i.e., based on considering each optical element in the scheme as a whole), some of the effects cannot be studied:

- Inhomogeneity of the fibers and optical elements
- Local bending of the fiber
- Local external parasitic effects

That is why it is advisable to divide each optical element into small layers with a thickness of Δz and follow the Jones differential matrices, which describe the transformation of the state of light polarization when it passes through a layer of small thickness. This is especially true for the sensor element, which is a coil made with a special spun fiber, since this fiber itself has a complex internal structure, and it is also necessary to take into account the bending of the fiber in the coil.

Thus, future research will seek to create a FOCS digital twin based on the model constructed using the Jones differential matrix formalism.

## 8. Patents

A.V. Medvedev, V.S. Temkina, A.V. Mayzel, “Demodulation method of fiber optic current sensor signal”, Patent RU № 2682981, G01R 15/22, G01R 15/24, G01R 19/00, Date of patent: Okt. 31, 2018.
